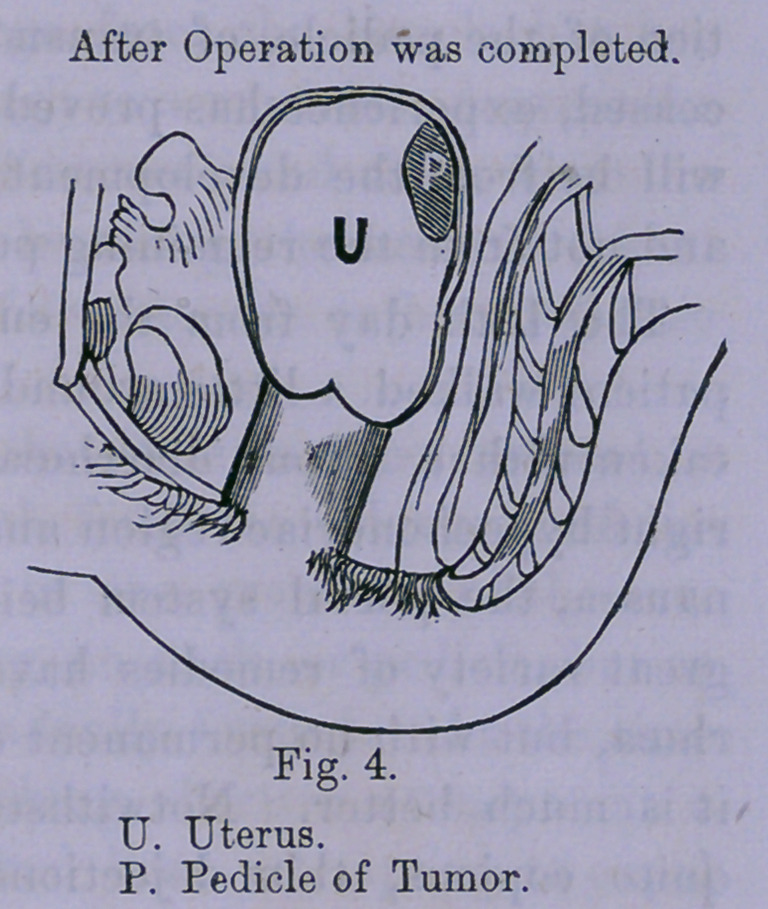# Fibrous Polypus of Uterus—Removal by Ligature

**Published:** 1867-11

**Authors:** G. W. Phillips

**Affiliations:** Dixon, Ill.


CHICAGO MEDICAL JOURNAL.

VOL. XXIV. NOVEMBER, 1867. No. 11.

### Original Contributions.

### FIBROUS POLYPUS OF UTERUS---REMOVAL BY LIGATURE.

By G. W. PHILLIPS, M.D., Dixon, Ill.

In order to make the report of the following case more intelligible, I shall premise it by reference to well-established facts, relating to the pathology and treatment of uterine tumors. These facts, being mainly obtained from the "Lectures of Dr. Charles West, on Diseases of Women," whose work contains the results of the collective observations, and experience of the best observers, upon the subject, and whose language I have freely quoted.

Ovarian dropsy, and tumors, as well as cancer of the uterus, need not be considered in this connection, but those less fatal, and non-malignant tumors of the womb, that affect some part of the body or cervix. "Hypertrophy of the uterus is more frequent than of any other organ, and each of its component tissues is liable to a similar overgrowth," constituting tumors. When the mucous membrane of the cervix becomes hypertrophied, small pendulous or sessile tumors are formed, composed wholly of an overgrowth of mucous membrane, called "mucous polypi." "If a larger quantity of cellular tissue enters into their composition," they become somewhat larger, and are "made up of fibro-cellular tissue, having an investment of cervical mucous membrane."

"More frequent than the last-mentioned tumors are those of a more complex character, into the formation of which there enter, not only the mucous membrane of the cervical canal, or its hypertrophied cellular tissue, but also the large mucous follicles of the cervix," and constituting the "glandular polypi."

Sometimes, these mucous follicles, or Nabothian glands, are hypertrophied, forming small "cysts," "without any outgrowth of the mucous membrane, or proper tissue" of the cervix. These growths, so far considered, are the simplest forms of uterine tumors, of small size, and almost always proceed from the cervical mucous membrane, and nearer the external than internal os, rarely springing from the mucous membrane of the body of the uterus. When the muscular walls, or proper tissue of the uterus, takes on an overgrowth, the *fibrous tumor* is produced, in "structure almost identical" with the uterine tissue—very frequent in their occurrence—may occupy any part of the exterior or interior of the womb, more frequently proceeding from the posterior wall, and are "rarely solitary, several tumors usually being present at the same time, one or two generally outstripping the others in their growth." They may be "developed immediately beneath the peritoneum that covers the uterus." These "superficial growths are generally limited to the fundus, more frequent on the posterior than anterior surface, and generally remain of small size;" may "proceed from the thickness of the uterine walls, and grow outward toward the cavity of the peritoneum, or inward toward the cavity of the womb, the former mode being the more frequent;" or may remain imbedded within the walls of the uterus, with no tendency to project outward or inward.

The tumors that grow outward are connected with the uterus by a thick pedicle, into which uterine fibers enter, but are not invested by uterine substance, like those that grow toward the cavity of the womb." "When they grow internally, they are sometimes positive outgrowths from the uterine tissue," their texture, and that of the uterus, being interwoven together, "and are often much more abundantly supplied with blood than the other varieties of these growths." The pedicle of the tumor is formed by uterine and dense cellular tissue, its proper coverings being the uterine mucous membrane, which envelops it completely, and a layer of uterine substance which, in some cases, invests it partially, and in others, completely, the mucous membrane and muscular tissue "undergoing development somewhat in proportion to the tumor;" such being its coverings, instead of "a fibro-cellular capsule of an ordinary fibrous tumor," and which constitutes that form of fibrous tumor called *fibrous polypus*.

The other varieties of fibrous tumors are distinct from the uterine tissue, whether imbedded in it or projecting from it, and are invested with a more or less complete "capsule of fibro-cellular tissue."

"Whatever the point of origin" of fibrous tumors, and whether they grow outward toward the cavity of the peritoneum, or inward toward the cavity of the womb, "they usually have a tendency to form a neck or pedicle," an important fact to be remembered.

"The influence which these growths exert upon the uterus is in proportion to the intimacy of the relation between the tumor and womb, rather than to the size to which the tumor attains." "When situated external to the womb, and growing into the peritoneal cavity," the tumor may acquire "an enormous size," and the uterus be perverted in shape, but little increased in size. A tumor imbedded and growing within the uterine walls developes the uterus similar to pregnancy. "The growths that project into the uterine cavity" produce a great increase in the size of the womb, not only from the expansion of its walls by the pressure of the growing tumor, but by an actual hypertrophy or growth of its substance.

Fibrous tumors sometimes exist, even of large size, without disturbing the functions of the organ at all. The constancy of the symptoms is generally proportionate to "the intimacy of the relation between the tumor and womb." "The growths which proceed from the outer surface of the womb often produce no symptom except such as may be due to their mechanical pressure upon adjacent organs, whilst those that are imbedded in the uterine substance almost always disturb the functions of the organ, even before they have attained any considerable size; and the polypi, or growths, which occupy the cavity of the womb, attract attention, almost from the first, by the hemorrhage they occasion;" the source of the hemorrhage was formerly thought to be from the tumor, or polypus, but is now considered to be from the irritated mucous membrane of the uterus.

There are several different methods by which nature attempts a cure, and sometimes cures fibrous tumors. That variety of fibrous tumor, that grows into the uterine cavity, has a "constant traction" exerted upon its pedicle, by the contractions of the uterus, gradually stretching and attenuating it, until it finally gives way, or is snapped off by the violent contractions of the womb; or, the margins of the os uteri may constrict the pedicle, and strangulate it, ligate it, as it were; or, "a portion of its investment," or covering, may ulcerate, and the tumor "gradually shell out"; or, "a change takes place in its substance," allied to softening, its tissue becoming gradually disintegrated, and flowing away as an offensive, dirty matter; this "is not a process of inflammation," with any of its results, but takes place without any symptoms of inflammation. I have met with one instance of the spontaneous cure of a fibrous tumor by this process. The tumor had existed for twenty years, the woman, for ten years, not being able to walk, and for two years, confined to her bed. An offensive discharge commenced to flow from the womb, and after a lapse of several years, the tumor disappeared, no doubt undergoing softening and disintegration. A process o softening, or death, often takes place "in the lower part of fibrous polypi, where they project through," or against "the os uteri"; "the mucous membrane covering it ulcerates, and thus being deprived of its most important source of nutrition, the adjacent surface of the tumor loses its vitality," and becomes disintegrated, the softening and death extending, perhaps, to the entire tumor; or, the tumor may undergo "the cretaceous transformation." These processes of softening and calcareous transformation are similar to what sometimes takes place in a tuberculous bronchial gland, or tubercle of the lungs. In those cases in which a cretaceous transformation of a fibrous tumor takes place, the growth has no longer a "vital relation" with the womb, consequently, the "derangements of function, which it produced, diminish or disappear"; in some rare instances, the calcareous mass is expelled. Tumors growing from the peritoneal surface, sometimes become spontaneously detached, the pedicle being, probably, absorbed, and the tumor becoming fixed near its point of origin, by "false membrane," its vital connection with the uterus having ceased. Fibrous tumors are, probably, never removed by absorption.

I have mentioned the methods by which nature sometimes effects a cure in cases of fibrous tumors. Art has several modes. Little or nothing can be expected from the use of medicine in these cases, except it may possibly be "to retard their growth" by the use of iodine, or bromide of potassium. It is only by surgical interference, that our art is of avail.

Those fibrous tumors that grow into the uterine cavity, having a neck, or pedicle, may be removed by excision, ligature, by the ecraseur, torsion, and by the "galvano-caustic wire."

The removal by ligature, has been the mode most commonly practiced, on account of the supposed less danger from hemorrhage, than by excision, yet experience has proved that the per cent of danger, from hemorrhage, by excision, is very small, even if the pedicle be large, fibrous tumors, or polypi, even having been "been cut away peicemeal," without dangerous hemorrhage. In the use of the ligature, there is more danger from purulent infection than by any other method, requiring several days to complete the operation, and subjecting the patient to greater inconvenience.

The operation, by means of the ecraseur, is a combined crushing and cutting process: a metalic ligature, like a chain saw, without teeth, or a wire rope, or cord, composed of many strands of annealed iron wire, twisted together, is carried around the neck of the tumor, then, by means of an apparatus to which the ligature is attached, and in which it works, drawn slowly and with great force, until the pedicle is cut and crushed off, thus obviating the small per cent of danger, from hemorrhage, attendant on excision, and rendering the danger from pyæmia very much less than by ligature. The method by torsion, or twisting, is only applicable to small polypi with slender pedicles.

The operation by means of the galvano-caustic wire, is a combined cutting and burning process: a platinum wire is passed around the pedicle of tumor, then heated, by a strong current of galvanism passed along it, the hot wire being dragged through the tissue by an appropriate handle. This method would seem to be preferable to all other modes, so far as danger from hemorrhage and pyæmia are concerned, but requires a battery of great power.

Those growths, that lie imbeded within the uterine walls, or grow outward into the peritoneal cavity, have, in some instances been removed, either "by enucleation through the os, or lower segment of the uterus," or by abdominal section, in some instances, the uterus being extirpated with tumor, but these operations have been attended with great fatality. Of late years, however, a greater proportion of successful cases have been reported of the extirpation of the uterus for fibrous tumor, by the knife, or the ecraseur, the latter mode being considered the better, mainly on account of the less liability to hemorrhage. One of the most remarkable operations of this kind, was recently performed by Dr. Storer, of Boston, a report of which was published in the *Amer. Jour. Med. Sciences*, for January, 1866.

Keeping in view these facts, as regards uterine tumors, the main points of interest in the following case will appear more clearly:

Mrs. H——r; age 41; dark-brown, reddish hair; fair skin; nervous, sanguine temperament. Previous history, eight years ago, soon after the birth of her last child, hemorrhage commenced from uterus, occurring not only at the monthly periods, but between them. I first examined her in the Spring of 1865, diagnosed a fibrous tumor growing within the uterine walls.

Her general health during this period has been poor, being anæmic from loss of blood, but still has kept about, except at those periods of the occurrence of hemorrhage, at which times she also suffered severe pain.

*December 31st, 1866.* For some time past, she has had, every two weeks, severe pains, the character of which have been more "like labor pains," but unattended with hemorrhage; in fact, the hemorrhage, for several months past, has been growing less. The cause of the cessation of the hemorrhage is not clear to me. Dr. Charles West, in his Lectures on the Diseases of Women, claims that the hemorrhage takes place from the uterine mucous membrane, and that "tumors hanging by a pedicle into the uterine cavity are attended by one invariable symptom, *viz.*, hemorrhage;" that it may cease suddenly if the tumor escapes into the vagina, or if its vital connection with the uterus ceases from those spontaneous changes, before alluded to; but in this case, none of those conditions occurred, yet the hemorrhage ceased. The abundant leucorrheal discharge, which has from first been a persistent symptom, has continued, mixed with debris from the tumor, the investing mucous membrane of which can be seen through the speculum to be ulcerated, at that part of the tumor that presses against the os, the adjacent part of the tumor having become softened, and disintegrated, an offensive, dirty, white discharge resulting. Os, dilated to the size of a silver half dollar, when uterus is not contracting, and rigid, closing tightly around the finger, when contraction comes on, the tumor being pressed firmly down against the os, filling up and engaged in the superior strait, as to size, feeling like a child's head at full term. The neck, or pedicle, feels as large as one's wrist, appearing to proceed from the posterior wall, and fundus of uterus; its attachment to the uterine walls cannot be clearly defined. I supposed, however, that I had ascertained the limit of the attachment inferiorally.

The base, or that part of the tumor presenting, is easily compressed, and has a very elastic feel, the neck is more firm. The tumor, nearly two years ago, being characterized by greater density, and hardness, and very much larger. The abdominal tumor extends as high as the umbilicus, occupying the space to the right, between the median line and crest of right illium.

She has obtained relief from the acute pain, by use of tinct. opii., taking f. ℥vj., in two hour's time. The pain from the violent contractions of the uterus is so severe, that the tinct. opii., even in these large doses, fails to give relief. I accordingly introduced within the os uteri, 15 grs. of the solid extract of opium, every six hours, which gave comparative relief from pain, and aided much in relaxing the os; at times, the pain was so severe, that chloroform was given by inhalation, in addition to the opium. After a few days had elapsed, when she had become comparatively free from pain, I commenced to dilate the os, by means of sponge tents, preparatory to removal of the tumor. This method of dilating the os, was, I believe, first brought to the notice of the profession by Prof. Simpson, of Edinburgh. In about ten days from the time I first commenced the use of the sponge tents, the os had become dilated to the size of a circle two inches in diameter, and soft and dilatable, the base of the tumor presenting within the os.

*January 15th.* It was now determined to remove the tumor. Appetite fair; pulse good. Drs. Everett and Law were called in, who rendered valuable assistance in the operation. The plan of operation, was to seize the tumor with the ordinary obstetrical forceps, as one would a child's head for delivery, bring it down beyond the vulva, or as low as possible, and excise it, as advised by Dr. Charles West, and other eminent obstetricians and surgeons; but in case this should prove to be impracticable, or very difficult, then to ligate it. The patient was placed upon her back, hips near the edge of the bed, legs and thighs flexed, and held apart by an assistant, chloroform administered, the forceps applied, the tumor, by steady traction, brought down, engaging in the inferior strait, rendering prominent and full the perineum, as a child's head would do, the base of the tumor appearing at the vulva, and, just as I expected, it was about to pass the inferior strait, the forceps slipped off, and the tumor partly returned within the uterus.

The forceps were reapplied, tumor brought down as before, when they again slipped off. They were applied a third time, and as the base of the tumor appeared at the vulva, a strong hook was thrust into it, and the tumor held down, as the forceps slipped off. The farther effort to bring the tumor beyond the vulva was abandoned for fear of producing inversion of the womb. The tumor, from its large size, filling up so completely the cavity of pelvs and uterus, making it difficult to use a cutting instrument, the plan of excising the tumor was abandoned, and a ligature applied around the supposed neck or pedicle of tumor, by means of Gooch's double canula.

The uterus became much reduced in size after the tumor was brought down, but the abdominal tumor still remained quite prominent, this was supposed to be accounted for by the large size of the pedicle, and the hypertrophy of the uterus, it was not thought that any part of the tumor was above the ligature, except the portion of the pedicle above, and attached to uterus. The tumor at the base was cleft, so as to form two lobes, pear-shaped, of a purple or dark raspberry color, elastic, easily compressed; it could not have been very vascular, as but a few drops of blood flowed, as the large, sharp hook was thrust deeply into its substance. The immediate constitutional effects of the operation were not severe; the pulse, during the operation, was full, and strong. She was now given, within two hours, f. ʒvij. of tinct. opii., and five hours after the operation, free from pain, skin warm, pulse full, a little sleepy—the tinct. of opii. was continued in f. ʒj. dose every two hours for twelve hours.

Three days before the operation, I commenced to give sulphite of soda, grs. x., every three hours, to guard against blood poisoning, which was continued until after the tumor had all sloughed away. Injections of a weak solution of chloride of soda were used as a disinfectant.

*January 17th.* Two days after the operation, she had a rigor; pulse 120 to 134, quick and weak; within an hour, the pulse became less frequent; not so weak; skin warmer, and perspiring. This attack was simply miasmatic, induced by the shock of the operation. Diet generous—oyster soup, soft boiled eggs, etc.

*January 20th.* Uterus contracted down to less than half the size it was before the operation, the tumor sloughing, and presenting outside of vulva, ligature still attached, and canula coming down with the mass. Upon examination, I find, that as the double-lobed mass passed out of the cavity of the pelvis, having shrunken to a-fourth of its original size, another mass or lobe, two-thirds the size of that ligated, had been expelled from uterus into the cavity of the pelvis, being harder, and less elastic, than the other mass. At the time of the operation, only that part of the tumor that was double-lobed, or the inferior section, (as shown by drawing No. 1,) was ligated, the upper

Before the Operation.

Fig. 1.

U. Uterus. T. Tumor.
B. Bladder. U. L. Upper Lobe.
V. Vagina. L. L. Lower Lobe.
P. Pedicle.

After Lower Lobe had been brought down into cavity of pelvis, by means of the obstretrical forceps, preparatory to ligating the neck of lower lobe.

Fig. 2.

U. Uterus. T. Tumor.
U L. Upper Lobe.
L. L. Lower Lobe.
P. Pedicle.

section, or third lobe, not having been brought down out of cavity of uterus, but after the inferior section had sloughed and shrunken, the contractions of the uterus expelled the upper section into cavity of pelvis, and the inferior section, outside the vulva. The inferior, or bi-lobed mass, was now drawn farther out of the vulva, and the neck excised, the ligature having nearly cut through, and the neck of the superior section, or lobe, ligated. It will be seen, (as the drawings show,) that the tumor consisted of two sections, and two necks, or pedicles, the superior mass being attached by a short, thick, firm pedicle, to the body and fundus of uterus, posteriorally, the inferior section being attached to the lower part of the superior, by a short, thick, less firm pedicle.

After removal of Cerva Lobe.

Fig. 3.

U. Uterus.

U. Upper Lobe after the lower was removed.

After Operation was completed.

Fig. 4.

U. Uterus.

P. Pedicle of Tumor.

*January 31st.* The 11th day from the application, the second ligature became loose, and the superior section of tumor, taken away. The entire mass of the tumor, before it had shrunken, would have weighed three or four pounds.

Immediately after the tumor was removed, I made, with the finger, a careful examination of that part of the pedicle remaining attached to uterus. The neck, or pedicle, was large, nearly or quite two inches in diameter, and having an extensive attachment to the body, and fundus of uterus, commencing at about the junction of cervix with the body, posteriorally, it was attached to posterior wall, forming a long thick ridge, running up to fundus, the limit of the attachment superiorally, I was unable to define clearly, as I was not able to reach to the depth of the uterine cavity. The attachment, inferiorally, was well defined, the pedicle feeling hard and smooth, the ligature having cut through the pedicle at this point within half an inch of the uterine wall; that part of the pedicle above was not so firm and smooth, but not elastic.

It will be seen by the drawings, that the ligature could not have been placed any higher, on account of the manner of the attachment of the pedicle. The considerable portion of the pedicle remaining, composed of uterine and dense cellular tissue, will become atrophied, and gradually disappear; the function of the pedicle, of transmitting blood to the tumor, having ceased, experience has proved that if another tumor occurs, it will be from the development of another and distinct growth, and not from the remaining portion of the pedicle.

The 12th day from the entire removal of the tumor, the patient walked a little around the room. At this time she was taken with a serous diarrhœa, with pain and tenderness over right hypochondriac region and ascending colon, with, at times, nausea, the portal system being in a state of hyperæmia. A great variety of remedies have been used, to check this diarrhœa, but with no permanent effect. At this date, August 6th, it is much better. Notwithstanding she would have a dozen quite copious, thin dejections in the twenty-four hours, she did not emaciate, but steadily gained in flesh and strength, keeping a good appetite; skin warm; veins full. I have, therefore, been led to the opinion that the diarrhœa is vicarious and conservative; that the system having been habituated to a daily drain of blood, or leucorrheal discharge, for years, when that drain was cut off by removal of the tumor, the congestion of the uterine system ceasing suddenly, that of the portal became congested, and took on the habit of depletion, which before was performed by the uterine system. The discharge from the uterus, a few days after the operation, became purulent, and continued in small amount a month. Five weeks from the complete removal of the tumor, the uterus had so lessened in size, that it could not be felt above the pubes; at this time, she had a neuralgic attack of liver, with pain in loins, with a bloody discharge from the uterus, (evidently the menses), which continued in small amount for a week; five weeks later she again had a menstrual discharge, and six weeks later, again; the fourth appearance of the menses did not occur, until seven weeks had passed. In this connection, I may mention, that during the continuance of the menstrual flow, the diarrhœa is much better, a fact going to show that the serous discharge from the bowels is vicarious.

Having for years been habituated to the use of opium, she finds it difficult to leave it off; in fact, she has had to take it, in order to moderate the discharge from the bowels, but her nervous system still requires, though to a much less degree, the stimulating effect of the drug, feeling weak and chilly when the effect wears off; she is gradually lessening the amount taken. Her general health is more than fair; attends to her domestic duties.

The history of this case shows that the tumor commenced to grow within, or was an outgrowth from the thickness of the uterine walls, and as it increased in size, grew toward the cavity of the womb, for a time having no neck, or pedicle, but as the growth increased, one was gradually formed. At the time of the operation, nature was making abortive attempts at a cure; the uterus, for some time, had been attempting to expel the tumor from its cavity, and had there been no interference, the organ would, eventually, have expelled the mass into the cavity of the pelvis, and, probably, a portion of it beyond the vulva; the softening and disintegration going on at the base of the tumor might, after a time, have extended to the whole of the tumor. The usual way, however, in which nature sometimes effects a cure in this class of fibrous tumors, or polypi, is in stretching and attenuating the pedicle, by the uterine contractions, until it finally gives way, or is snapped off by the violent efforts of the uterus. The prospect of nature effecting a cure by this means, was very doubtful, as the pedicle was so large and firm.

This was a case in which the operation by the ecraseur might have been practiced, and had I appreciated the value of the instrument, as I now do, I should have used it.

## Figures and Tables

**Fig. 1. f1:**
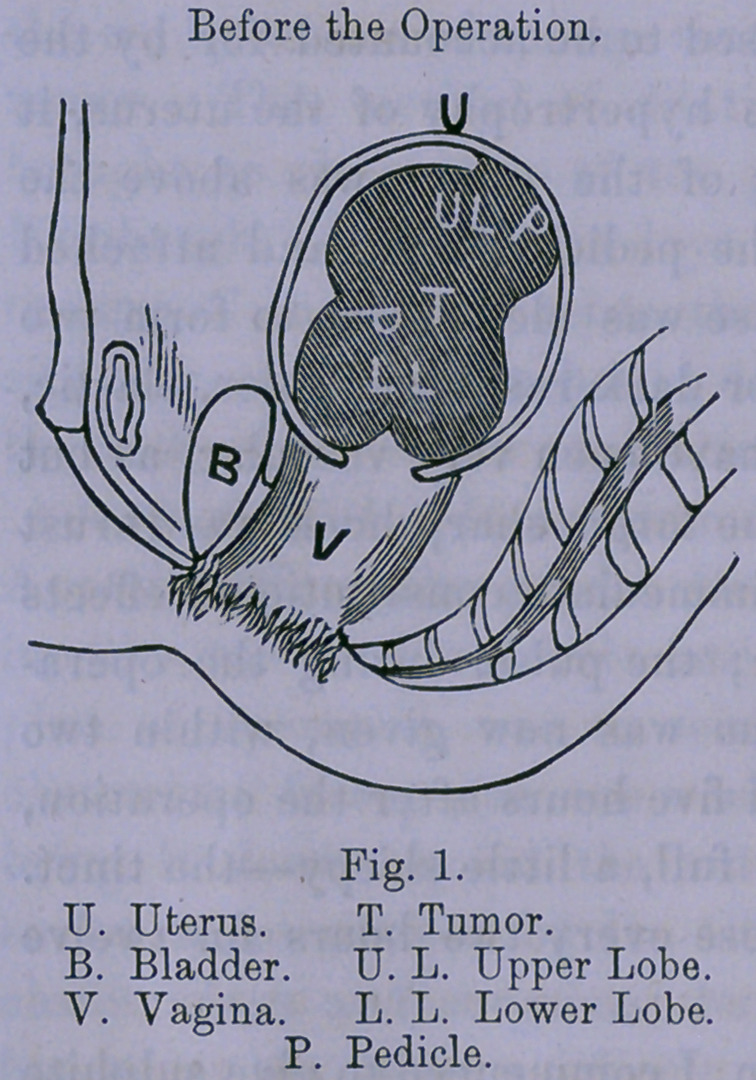


**Fig. 2. f2:**
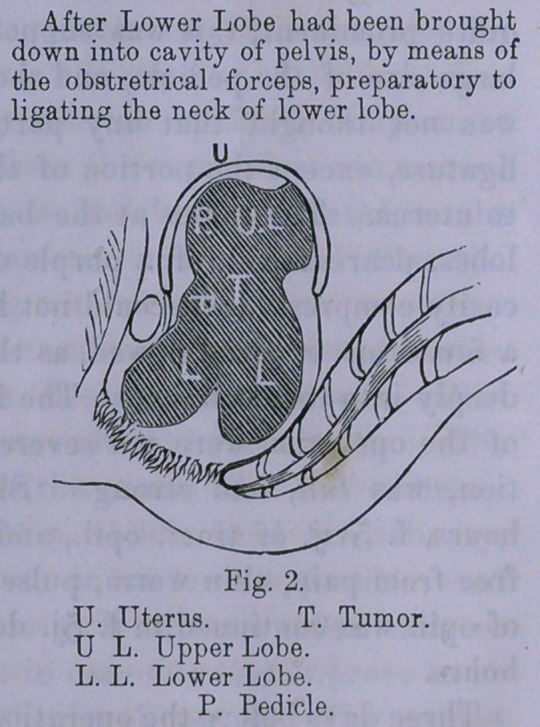


**Fig. 3. f3:**
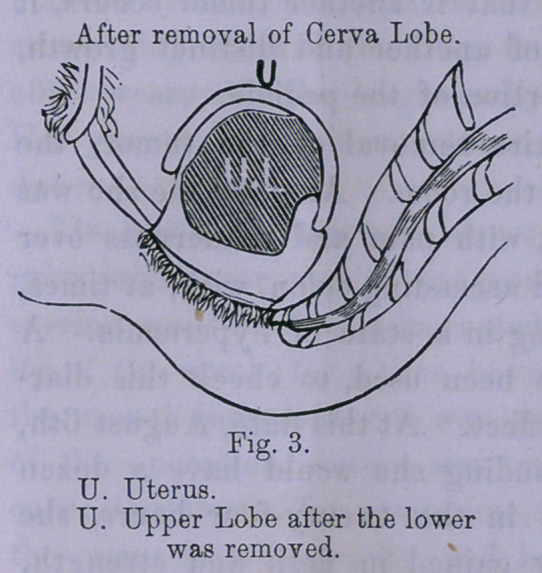


**Fig. 4. f4:**